# Influence of the Presence of B Chromosomes on DNA Damage in *Crepis capillaris*

**DOI:** 10.1371/journal.pone.0087337

**Published:** 2014-01-27

**Authors:** Jolanta Kwasniewska, Agnieszka Mikolajczyk

**Affiliations:** Department of Plant Anatomy and Cytology, University of Silesia, Katowice, Poland; Leibniz-Institute of Plant Genetics and Crop Plant Research (IPK), Germany

## Abstract

The sensitivity of different plant species to mutagenic agents is related to the DNA content and organization of the chromatin, which have been described in ABCW and bodyguard hypotheses, respectively. Plant species that have B chromosomes are good models for the study of these hypotheses. This study presents an analysis of the correlation between the occurrence of B chromosomes and the DNA damage that is induced by the chemical mutagen, maleic hydrazide (MH), in *Crepis capillaris* plants using comet assay. The presence of B chromosomes has a detectable impact on the level of DNA damage. The level of DNA damage after MH treatment was correlated with the number of B chromosomes and it was observed that it increased significantly in plants with 3B chromosomes. We did not find evidence of the protective role from chemical mutagens of the constitutive heterochromatin for euchromatin in relation to DNA damage. The DNA damage involving the 25S rDNA sequences was analyzed using the comet-FISH technique. Fragmentation within or near the 25S rDNA involved the loci on the A and B chromosomes. The presence of B chromosomes in *C. capillaris* cells had an influence on the level of DNA damage that involves the 25S rDNA region.

## Introduction

Numerous factors determine the genetic effect that is observed after mutagenic treatment, including the ability of mutagens to penetrate the tissue, cells and nucleus; the concentration of the mutagen and the treatment time [1]. The somatic and genetic effects differ in different plant species and as well as within different varieties. The sensitivity of different plant species to mutagenic agents appears to be related to their nuclear volume and DNA content [2]. The amount and type of chromatin in the nucleus may affect the induced mutations rates. Two hypotheses are related to these relationships. The bodyguard hypothesis proposes that a constitutive heterochromatin may protect euchromatin from chemical mutagens [3]. Heterochromatin localizes during interphase near the nuclear membrane which can absorb environmental stimuli more than euchromatin and thus it may protect the DNA from being mutated. The ABCW hypothesis states that the mutation rate per locus is proportional to the DNA content of a species [4], [5].

B chromosomes, also called supernumerary chromosomes, are present in hundreds of plant and animal species [6], [7]. They have been a subject of interest for many years, but their function has not yet been determined [8]. Whether B chromosomes carry genetic information or whether they are parasite chromosomes are still open questions. In some species, B chromosomes can carry ribosomal RNA genes. Moreover the transcriptional activity was shown for B-located ribosomal RNA genes in *Crepis capillaris* [9]. *C. capillaris* is one of the species with a different number of B chromosomes, from zero to five. Moreover, this species is characterised by a small number of chromosomes (2n = 6) and a favorable number and localization of rDNA sites. In addition to the NOR on chromosome 3 which contains about 2500 rRNA cistrons, the two telomeres of each B chromosome are estimated to carry about 400 rRNA cistrons [10]. A *Crepis* bioassay has been shown to be an excellent plant bioassay for assessing the chromosome damage that is induced by chemicals and environmental pollutants [11]. Predictably, the presence of B chromosomes increases the nuclear DNA content of plant cells. One B chromosome increases the amount of DNA by 6.8% and the mean area of the nuclei by 27.6% in *C. capillaris* cells. The amount of heterochromatin in B chromosomes can vary substantially in different species. Heterochromatin occupies 35% of the B chromosomes in *C. capillaris* [10] and 75% in *Zea mays* L. [12], while it has not been detected in some species (e.g., *Allium flavum*) [13].

The plant species that are characterized by the presence of B chromosomes are good models for the analysis of the ABCW and bodyguard hypotheses. A comparison of the frequencies of spontaneous and ethane methyl sulfonate (EMS)-induced mutations at the *yellow-green2* locus and/or deletions of this locus in maize plants with 0 or 4 B chromosomes was applied in order to evaluate these hypotheses. A significantly higher mutation frequency was found in plants that contained 4B than in plants with 0B chromosomes, by determining the frequency of mutant sectors on leaves, thus indicating that cells may be more susceptible to mutagens when B chromosomes are present [12]. Nuclear cleavage is the crucial DNA damage that is induced by chemical and physical mutagens. The comet assay has been widely accepted as a reliable method to detect for such DNA damage [14]. It is a method that is used to analyze genomic DNA damage and repair by measuring the level of single- and double-strand DNA breaks and alkali-sensitive sites in individual cells. The direct effect of maleic hydrazide (MH) on *C. capillaris* DNA has previously been shown using comet assay [15]. The comet-FISH technique permits the localization of a specific chromosome, regions of chromosomes or specific genes within the comets [14]. Thus, it allows DNA damage and the repair rate within the specific DNA sequences and their sensitivity to various damaging agents to be measured.

B chromosomes may have an impact on organisms, namely on the condensation of chromatin, transcriptional activity, recombination of A chromosomes and the induction of chromosomal aberrations [16], [17]. The latest work showed that B-located pseudogene-like fragments have the potential to regulate the expression of A-located genes [18]. However the influence of the presence of B chromosomes on DNA damage observed as DNA breaks has not previously been shown.

This study presents an analysis of the correlation between the occurrence of B chromosomes and the DNA damage that is induced by a chemical mutagen. Using comet assay, we analysed the level of nuclear DNA damage in the model plant species, *C. capillaris*, which had a different number of B chromosomes from 0 to 3. The DNA damage involving the 25S rDNA sequences was also analyzed using fluorescence *in situ* hybridization (FISH) applied on the comet slides (comet-FISH). MH, a clastogen commonly applied in plant mutagenesis to induce DNA breakage and chromosome aberrations, was used as the mutagen.

## Material and Methods

### Material and treatment

Seedlings of *C. capillaris* (2n = 6) were cultivated in a plant growth chamber at 21°C to the 4- 5 leaf stage. The number of B chromosomes was analyzed in the young leaf cells. In order to accumulate metaphases, leaves were treated with 2 mM 8-hydroxyquinoline for 2 h at room temperature and 2 h at 4°C, and then fixed in methanol-glacial acetic acid (3∶1). Chromosome preparation was done using the standard protocol for the enzymatic maceration of plant material. Leaves were digested with an enzyme mixture containing 2% cellulase (Onozuka, Serva) and 20% pectinase (v/v; Sigma) for 1 h at 37°C, washed in citric buffer (pH 4.7) and squashed in 45% acetic acid. After the removal of the coverslips on dry ice, slides were dried. DAPI (2 µg/ml, Sigma) chromosome staining was then applied.

The roots were immersed in 1 or 2 mM maleic hydrazide (MH, CAS no. 123-33-1, Sigma) solution in distilled water for the treatment. The plants were treated at 21°C for 4 h. The control plants were placed in distilled water. The roots were washed three times for 5 min in distilled water. The nuclei were isolated from the leaves immediately after treatment.

### Comet assay

The procedure for the comet assay was as described by Gichner and Plewa [19] with slight modifications. MH (CAS no. 123-33-1), normal melting point (NMP) and low melting point (LMP) agaroses and general laboratory reagents were purchased from Sigma Chemical.

After the seedlings were treated, individual leaves were placed in a small Petri dish on ice and spread with 200 µl of a cold 400 mM Tris-HCl buffer, pH 7.5. Each leaf was gently sliced into the “fringe” to release nuclei into the buffer using a fresh razor blade. Each slide, which had previously been coated with 1% NMP agarose and dried, was covered with a mixture of 55 µl of nuclear suspension and 55 µl of LMP agarose (1% prepared with phosphate-buffered saline) at 40°C and coverslipped. The slides were placed on ice for at least 5 min and then the coverslips were removed. Then, 110 µl of LMP agarose (0.5%) was placed on the slides and the coverslips were mounted once again. After 5 min on ice, the coverslips were removed. The slides with nuclei were placed in a horizontal gel electrophoresis tank containing a freshly prepared cold electrophoresis buffer (300 mM NaOH, 1 mM EDTA, pH>13) and incubated for 15 min. Electrophoresis was performed at 15 V, 340 mA for 15 min at 4°C. The electrophoresis conditions used in the study were optimal as they proved to provide low level of DNA damage in the control cells and a linear concentration-response for the induction of comets after chemical mutagenic treatment in these species in earlier studies (data not presented). The gels were then neutralized by washing them three times for 5 min in 400 mM Tris-HCl, pH 7.5 at room temperature and stained with 40 µl DAPI (2 µg/ml).

Fifty randomly chosen cells from each slide were analyzed under a fluorescence microscope with an excitation filter of 546 nm and a barrier filter of 590 nm using computerized image analysis system (Komet Version 5.1. Kinetic Imaging, Liverpool, UK). The % tail DNA was used as the parameter of DNA damage. The median values of % tail DNA were calculated for each slide and then the average median % tail DNA was calculated for each treatment group.

Each experiment was repeated twice and three slides were analyzed per each experimental group. No differences were observed for results from repeated experiments so a total of 300 cells of each experimental group were analyzed. From the repeated experiments, the average values of SD of each parameter for each treatment group were calculated from the median values from each slide. Data were analysed using OriginPro 8.5.1 software (OriginLab Corporation, Northampton, MA). The mean values of % tail DNA were analysed using a one-way ANOVA test. If a significant F-value of P<0.05 was obtained, a Dunnett's pairwise comparison test was performed.

### Fluorescence *in situ* hybridization (FISH) on comets

FISH was applied to the comet slides that had previously been analyzed for DNA damage, according to the method described by Kwasniewska et al. [15] with some slight modifications. The nuclei on the gels were denatured for 30 min at room temperature using a solution of 0.5 M NaOH and 1 M NaCl. Then, the gels were washed four times for 5 min in dH_2_0 and neutralized in 0.4 M Tris-HCl for 30 min at room temperature. The denaturized DNA was dehydrated in an ethanol series (70, 90, 100%, 5 min each) and the gels were air dried. 25S rDNA that was isolated from *Arabidopsis thaliana* [20] and labelled with digoxygenin-11-dUTP using nick translation (Roche) was used as the DNA probe. The hybridization mixture, containing 2.5 µg mL–1 of labelled DNA, 50% (v/v) formamide, 10% (w/v) dextran sulphate and 0.1 mg µL–1 salmon testes DNA in 2× SSC, was denatured at 75°C for 10 min and immediately placed on ice for a few minutes. The hybridization mixture (40 µl) was dropped on each gel, covered with a coverslip and incubated for 24 h at 24° in a wet-chamber. Before detection of the probes, the slides were washed for 10 min in 2x SSC, RT and 10 min in 0.1x SSC, RT. The digoxigenin-labelled probes were detected using FITC-conjugated anti-digoxigenin antibodies (Roche). After dehydration in an ethanol series, the slides were mounted in a Vectashield medium (Vector) containing 6 µg mL–1 DAPI. Preparations were examined using an OLYMPUS PROVIS epifluorescence microscope equipped with the proper filter set. Images were captured using a Hamamatsu C5810 CCD camera and processed in Adobe Photoshop 4.0. The localization of the 25S rDNA signals (comet head or comet tail) were recorded for each nucleus with a tail. Of the comets with a tail that were examined, four categories were distinguished based on the distribution of FISH signals between the head and the tail. Thirty comets per slide were analyzed. Each experiment was performed three times and then five slides were analyzed per each experimental group. The data on frequencies of comets belonging into specific categories were analyzed using a nonparametric Kruskal–Wallis one-way analysis of variance by rank test. If a significant K-value of P<0.05 was obtained, a Mann– Whitney U test within all categories of comets was carried out.

## Results

**Figure 1.Full:**
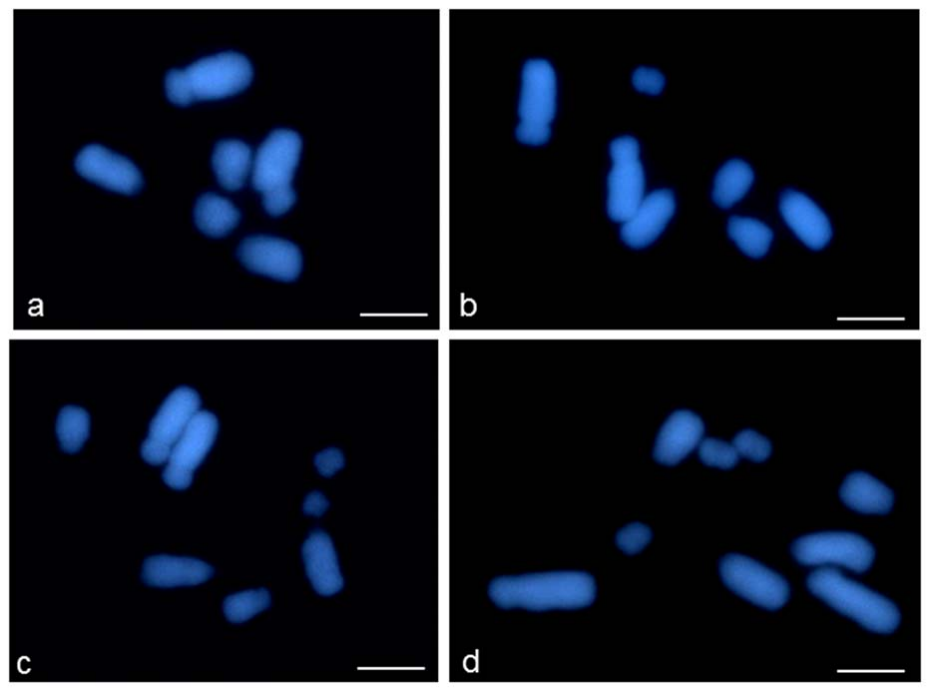
Full metaphase chromosome complements of *Crepis capillaris* with a different number of B chromosomes (DAPI staining). a) 2n = 6+0B; b) 2n = 6+1B; c) 2n = 6+2B; d) 2n = 6+3B. Bars represent 5 µm.

In this study the level of total DNA damage and damage near/within the 25S rDNA in *C. capillaris* cells with a different number of B chromosomes from 0 to 3 was evaluated (Fig. 1).

**Figure 2:**
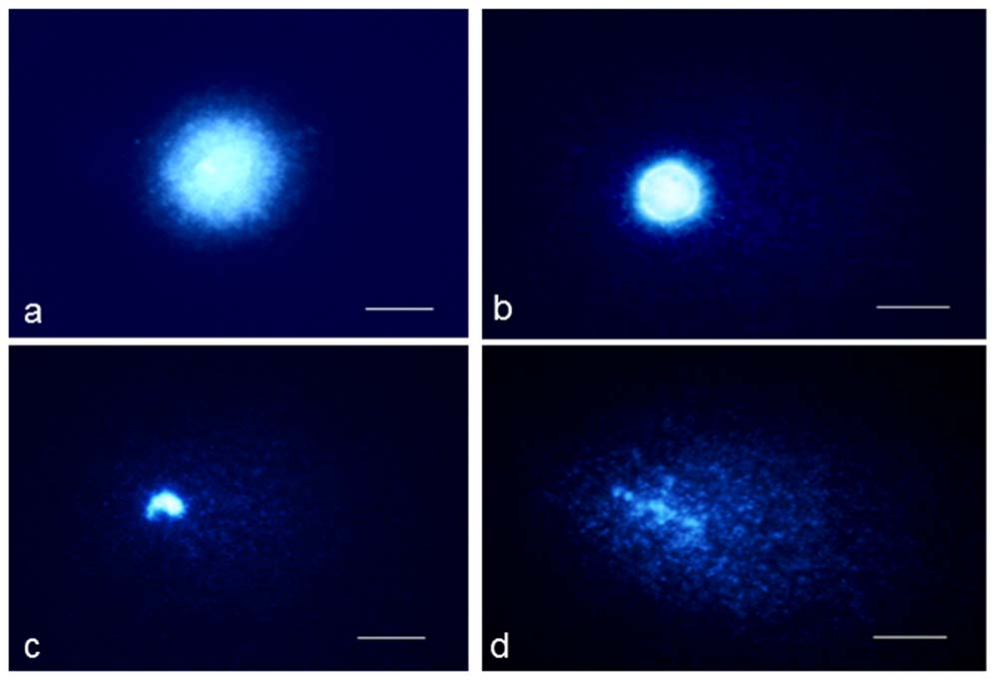
Comet assay in *C. capillaris* cells. a – control nuclei, not damaged; b, c, d – cells treated with a 2 mM MH, nuclei with different levels of DNA damage. Bars represent 5 µm.

**Figure 3:**
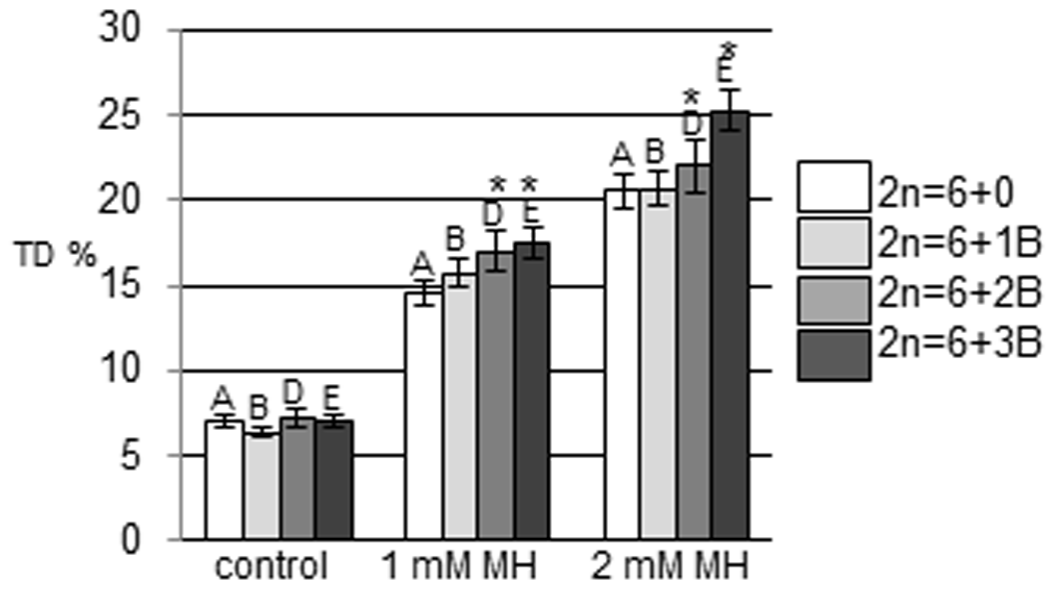
DNA damage (% tail DNA) in control and MH treated cells with a different number of B chromosomes. The bars indicate standard errors (SE).) The capital letters A, B, D, E indicate that the % tail DNA values after treatments are significantly different (P<0.05) from the % tail DNA of the control groups for the cells with the same number of B chromosomes. An * indicates that the % tail DNA values in 0B cells are significantly different (P<0.05) from the % tail DNA in cells with B chromosomes within the same treatment.

The majority of the control nuclei demonstrated a very low level of DNA damage – comets without a tail or with a short tail were observed. The level of DNA damage expressed as tail DNA that was detected in the control cells was similar in all of the *C. capillaris* lines – from 6.38 to 7.23%. The treatment with 1 or 2 mM MH led to DNA damage, which was observed as comet formation in all of the *C. capillaris* lines (Fig. 2). The values of the % tail DNA were higher than in the control after MH treatment (Fig. 3). The treatment with 2 mM MH led to a significantly greater increase in the level of DNA damage than 1 mM MH. The level of DNA damage induced by MH was different for the *C. capillaris* lines that were analyzed and were dependent on the number of B chromosomes. The highest level of DNA damage was observed in the lines with 3 B chromosomes; the lowest – in the cells without B chromosomes. The % tail DNA after 1 MH treatment was 14.5% in the 0B lines and 17.5% in the cells of 3B lines. Even greater differences in the level of DNA damage was observed after 2 mM MH treatments. The level of DNA damage induced by 2 mM MH expressed as % tail DNA was about 20.6% in the 0B lines and was significantly higher in the 3B lines – 25.3%. These results indicate the higher susceptibility of the *C. capillaris* plants to the chemical mutagen, MH, when B chromosomes are present. The presence of B chromosomes always has a detectable impact on the level of DNA damage; however, this seems to be correlated with the increased number of B chromosomes, rather than being an odd-even effect. Namely, the level of DNA damage significantly increased in plants containing 3B chromosomes after MH treatment.

Comet-FISH with 25S rDNA probes was used to analyze the number and distribution of these sequences in *C. capillaris* in control and MH-treated comets. We analyzed whether the presence of B chromosomes had an influence on the damage involving the 25S rDNA. FISH signals were assigned to the damaged or undamaged part of the comet (tail and head, respectively). The presence of a higher number of dots in the comet tail after MH treatment than in the control cells was observed, thus indicating that rDNA sequences are present in small fragments.

**Figure 4:**
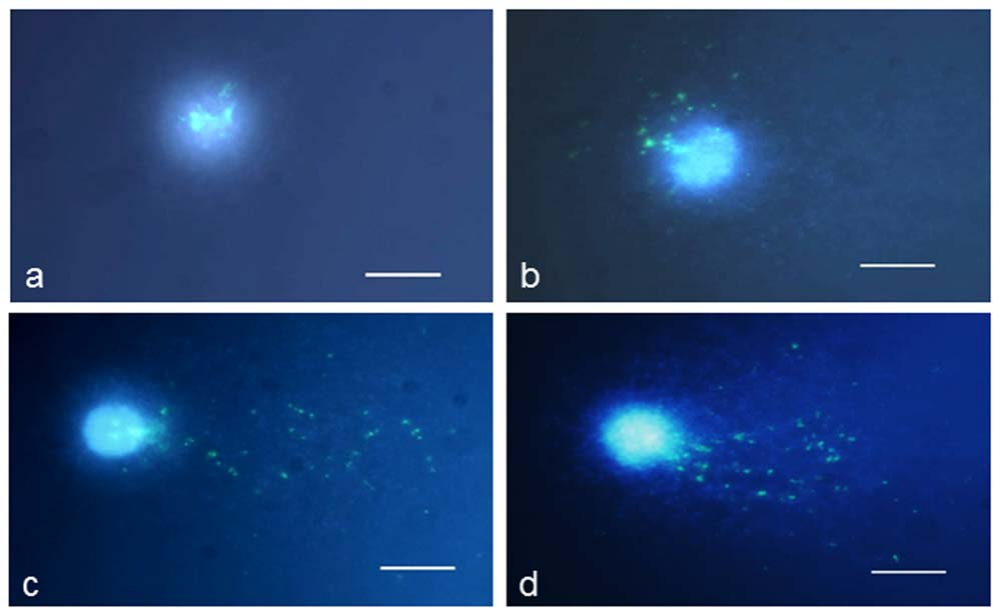
Examples of control and MH-treated nuclei with the distribution of 25S rDNA FISH signals. Bars represent 5 µm.

**Figure 5:**
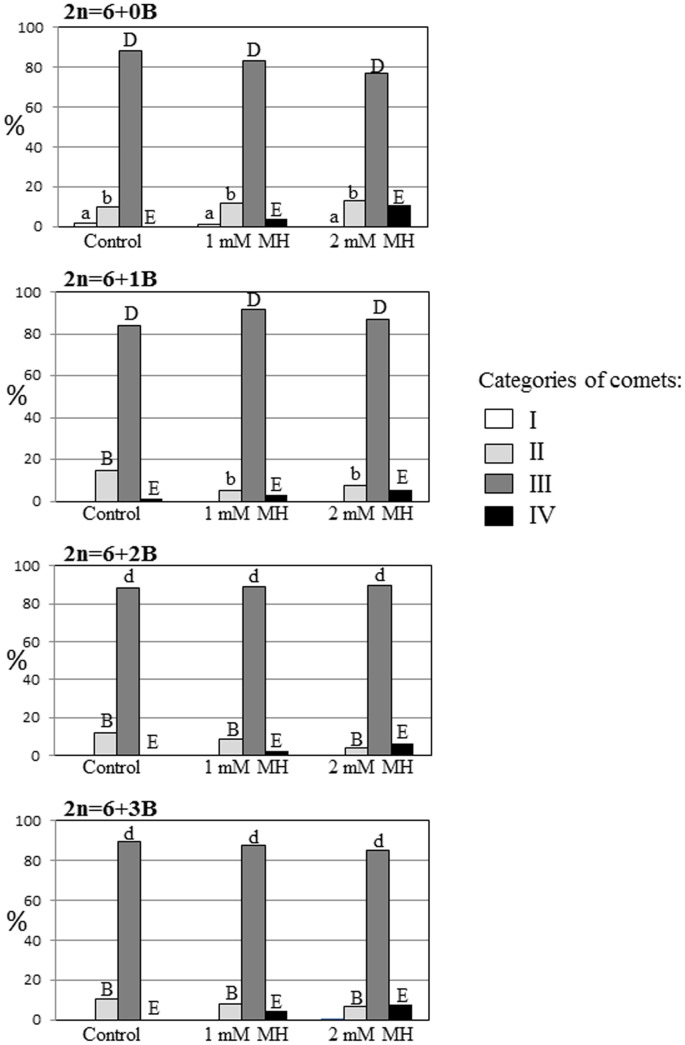
The frequencies of comets classified as category I to IV in control and MH treated cells. The capital letter indicates that the frequency of the comets is significantly (P<0.05) different from the frequency of comets of the same category from other experimental groups (control, 1 mM MH, 2 mM MH) in *C. capillaris* cells with the same number of B chromosomes. The small letter indicates the frequency of the comets is not significantly (P>0.05) different from the frequency of comets of the same category from other experimental groups (control, 1 mM MH, 2 mM MH) in *C. capillaris* cells with the same number of B chromosomes marked by the same small letter.

Four categories of comets in the control and MH- treated cells were distinguished based on an analysis of the distribution of FISH signals between the head and the tail (Fig. 4). Comets with 25S rDNA signals in the head are classified as category I (Fig. 4a), while comets with signals in the heads and the ‘halo’ are classified as category II (Fig. 4b). Comets with 25S rDNA in the head, halo and tail are classified as category III (Fig. 4c). Comets representing category IV are characterized by the total migration of 25S rDNA to the ‘halo” and to the tail (Fig. 4d). We analyzed the frequencies of each category of comets with a specific distribution of 25S rDNA signals (Fig. 5). The same categories of comets were observed for the control and MH-treated cells; however differences in the frequencies of the categories were observed.

Comets in category III are observed most often in non-treated and MH-treated cells – about 80% for all of the *C. capillaris* lines. Comets in category I were observed only in the 0B line, in the control and in the 1 mM MH treated cells. No comets in category IV were observed in the control cells, although they were observed after MH treatment and their frequencies increased with the concentration of the mutagen. No differences were observed regarding the comets in category IV in the C. *capillaris* lines that were analysed. The frequencies of comets in category II did not change after MH treatment only in cells of 0B line. In contrast to the 0B line, the frequencies of comets in category II in the plants that contained B chromosomes significantly decreased after the treatment with MH.

## Discussion

In this study we evaluated the ABCW and bodyguard hypotheses in relation to direct DNA damage. The presence of supernumerary chromosomes in *C. capillaris* is associated with increase in DNA damage induced by the chemical mutagen, MH. The presented results support the idea of the ABCW hypothesis that there is a correlation between the level of DNA damage and DNA content in somatic cells. This hypothesis has previously been proven by the direct correlation between the frequency of radiation and EMS-induced mutations and the DNA content in a nucleus in maize [6], [12]. The distinguishing between A and B chromosomes was not possible so we could only conclude that presence of B is associated with increase in DNA damage in presence of mutagen. Although the concept of the ABCW hypothesis has been proven, it should be mentioned that it has also been the subject of controversy [21]. This study did not support the idea of the protective role of the constitutive heterochromatin for euchromatin from chemical mutagens. The presence of the B chromosomes increases the total amount of the heterochromatin in *C. capillaris* cells [22], however it is not protecting from DNA damage. However it needs to be emphasized that the amount of heterochromatin of B chromosome in *C. capillaris* is similar to A chromosome. Similarly, the experimental results using EMS and ionizing radiation in maize are in disagreement with this hypothesis [12]. The protection of the coding regions of a genome is one of the possible functions of the heterochromatin that represents non-coding DNA, and which is often described as junk DNA [23]. It forms a layer on the outer surface of the nucleus. As an example, heterochromatic blocks around a secondary constriction may ensure against any evolutionary change of ribosomal cistrons by decreasing the frequency of crossing-over in meiosis and absorbing the effects of mutagens. Hsu [4] stated that the heterochromatin is effective in protecting the euchromatin against chemicals, but not against radiation.

Studies using a comet assay on the sensitivity of different plant systems to mutagenic agents that have been analyzed as DNA breakage are well known. The comet assay is a sensitive method for assessing DNA damage in genotoxicity testing and fundamental studies on mechanisms of DNA damage and repair [14]. The optimization of the comet assay procedure for plant tissue permits the comparative analysis of a wide range of DNA-damaging genotoxic agents in numerous plant systems [24]. The role of various cellular components in mutagenesis processes has been evaluated using the comet assay [25], [26]. The sensitivity of the nuclei of different species depends on the type of mutagen that is used for the induction of the damage. *Allium cepa* and *Nicotiana tabacum* cells are equally sensitive to the genotoxic stress that is induced by peroxide and the heavy metal cadmium, and an increased sensitivity of the cell nuclei in A. *cepa* after treatment with EMS has been reported [27]. The level of the chromatin condensation, which reflects the proportion of heterochromatin/euchromatin, could be responsible for the differences in the sensitivity of different species [28]. The sensitivity of euchromatin and heterochromatin to mutagens is different and therefore the behaviour of the different DNA sequences under comet assay conditions is not the same [29]. However, the differences in the sensitivities of 5S and 25S rDNA that were previously reported were explained by the association of the active 25S rDNA with the nucleolus [15]. Chromosomes with a high gene density are more resistant to DNA damage or have a very effective repair system [30].

B chromosomes are not found in all species and they vary in size and number among species. They are not important for survival and may be thought of as parasitic elements. B chromosomes are the source of intraspecific variation in the amount of nuclear DNA in numerous plant species [31]. As their presence increases the genome size, the differences in the sensitivity of plant cells to mutagens with B chromosomes may be correlated with this fact.

The changes in the organization of chromatin that is caused by the presence of B chromosomes may influence the DNA damaging effect of the MH treatment that was observed in this study. The DNA composition of B chromosomes is similar to that of A chromosomes. However, the B chromosomes in *C. capillaris* are extensively heterochromatic – they display a centromeric-pericentromeric band covering 35% of the total length [32]. The presence of active 25S rRNA was observed not only at the nucleolus organizer of both No. 3 chromosomes but also at the distal segments of all of the B chromosomes [11]. The greatest sensitivity of *C. capillaris* plants was proven when 3 B chromosomes were observed in this study. The presence of B chromosomes in somatic cells also influenced the sister chromatid exchange (SCE) frequency in A chromosomes; however, only when they were present in odd numbers [31]. There is still no explanation for this strange odd-even effect. There was no odd-even effect on the level of DNA damage in our study.

The different sensitivity of the cells with and without B chromosomes to mutagens may be correlated with the methylation level. Differences in the DNA methylation level the A and B chromosomes of *C. capillaris* have been shown [33]. However, B chromosomes showed the same pattern of methylation of histone H3 as standard chromosomes [16]. An increase in the amount of DNA due to the presence of B chromosomes affects the duration of the cell cycle. The duration of the cell cycle and DNA synthesis is not directly proportional to the number of B chromosomes. The ratio of the increase that is caused by B chromosomes is greater than could be expected on the basis of the effect obtained by the additional A chromosomes [34]. As the duration of the cell cycle and DNA synthesis is extended, more cells could be subjected to MH, which is an S-dependent mutagen [35].

Comet-FISH was used to show the effects of B chromosomes on the involvement of 25S rDNA sequences in comet formation. The analysis of the distribution of these DNA sequences was applied by classifying the comets into categories. An automated analysis was not possible as the number of minute signals was high andmany of the signals were observed as chains. This indicates that at least part of the fragmentation occurs within the 25S rDNA sequences. Due to the differences in the frequencies of comet categories in the *C. capillaris* lines with a different number of B chromosomes, we conclude that fragmentation within/near the 25S rDNA involved the loci on A and possibly B chromosomes. The analysis of chromosome aberrations using FISH with 25S rDNA as probe in *C. capillaris*, showed that B chromosomes are involved in their formation in addition to A chromosomes [17].

The frequencies of comet categories I and II did not change after treatment with the MH only in the cells without B chromosomes. This may indicate the higher stability of 25S rDNA in *C. capillaris* cells without B chromosomes. The mechanisms of differences in the sensitivity of cells with B and without B chromosomes to mutagens without distinguishing between A and B chromosomes remain unknown, however few theories could explained them. The lack of changes in the housekeeping genes, which are represented by 25S rDNA, is crucial for the cells, in which only one chromosome pair in diploid cell is marked by 25S rDNA using FISH. It is possible that 25S rDNA in A chromosomes is protected. Rapp et al. [30] found that chromosomes with the highest density of active genes were most likely not be subjected to fragmentation. Such phenomena could be an argument for the higher stability of the 25S rDNA in A than in B chromosomes. A chromosomes are characterized by a higher gene density than B chromosomes. Moreover, the 25S rRNA genes of B chromosomes are only weakly transcribed [32]. The influence of B-chromosomes on the organization of rDNA in rye that is based on the compaction changes of the rDNA of A chromosomes was observed [36], [37]. The more condensed chromatin within the 25S rDNA region in A than in B chromosomes as well as the changes in the localization of the NOR region in interphase cells, which is caused by the presence of B chromosomes could make A-located 25S rDNA less sensitive to mutagens than B-located one. It is also possible that breaks between the centromere and the 25S rDNA sequences gives rise to a spot in a comet. As 25S rDNA sequences are relatively close to the centromere on chromosome 3 and a much greater distance from the centromeres on B chromosomes, the fragments would be generated more frequently when B chromosomes are present.

In summary, this study proves that the presence of B chromosomes may increase the sensitivity of somatic cells to DNA damage that is induced by chemical mutagen. We also showed a higher stability of 25S rDNA in *C. capillaris* cells without B chromosomes than cells with B chromosomes.
